# What do we know about pathological mechanism and pattern of lung injury related to SARS-CoV-2 Omicron variant?

**DOI:** 10.1186/s13000-023-01306-y

**Published:** 2023-02-10

**Authors:** Roberto Scendoni, Mariano Cingolani

**Affiliations:** Department of Law, Institute of Legal Medicine, University of Macerata, Piaggia Dell’Università, 2, 62100 Macerata, Italy

**Keywords:** SARS-CoV-2 variants, Omicron, Immune escape process, Lung injury, Pathological pattern, Diffuse alveolar damage, Ventilator-induced lung injury, Comorbidity, Autopsy

## Abstract

Pulmonary damage in SARS-CoV-2 is characterized pathologically by diffuse alveolar damage (DAD) and thrombosis. In addition, nosocomial bacterial superinfections and ventilator-induced lung injury (VILI) are likely to occur. The SARS-CoV-2 Omicron variant have manifested itself as a more diffusive virus which mainly affects the upper airways, such as the nose and pharynx. The mechanism leading to a lung injury with a complex clinical course for the Omicron SARS-CoV-2 variant remains unclear. A key question is whether the organ damage is due to direct organ targeting of the virus or downstream effects such as an altered immune response. An immune escape process of Omicron variant is being studied, which could lead to prolonged viral shedding and increase hospitalization times in patients with comorbidities, with an increased risk of pulmonary co-infections/superinfections and organ damage. This brief commentary reports the current knowledge on the Omicron variant and provides some useful suggestions to the scientific community.

## Comment

As SARS-CoV-2 continues to spread and cause diseases, emerging variants of the virus are being identified around the globe. The persisting challenges of SARS-CoV-2 to the international public health system have elicited concerns among scientists, drug and vaccine developers and the general population [1]. Since the beginning, five variants of concern (VOC) with increased transmissibility, varied virulence, evasion from therapeutic drugs, or decreased effectiveness of vaccines have been reported after the first one: Alpha, Beta, Gamma, Delta, and Omicron.

The Omicron variants have manifested themselves as a more diffusive virus which mainly affects the upper airways, such as the nose and pharynx, sparing the lower airways where the "more serious" pathologies occur and which often involve complex hospital management [2].

Numerous research groups around the world have thus begun to investigate in the laboratory, through in vitro and in vivo studies, the different characteristics of Omicron compared to previous variants, such as Delta. The Omicron variants are the most heavily mutable among all the VOC so far, which paves the way for enhanced transmissibility and partial resistance to immunity induced by COVID‐19 vaccines [3].

What emerges is a difficulty of Omicron to replicate efficiently in the lung tissue: studies support the reason it could lie in the less effective interaction with the Transmembrane Serine Protease 2 (TMPRSS2) protein, present on the surface of many lung cells and which helps the virus to evade their defenses [4]. The TMPRSS2 protein, on the other hand, would not be present on nose and throat cells: this could explain why Omicron performs better in the upper airways, where it has a high viral load [5]. This entry pathway switching is accompanied by alterations in proteolytic processing and reduced syncytia formation in infected cells, likely to limit cell-to-cell transmission and pathogenesis.

Scientific literature [6] has demonstrated how SARS-CoV-2 induced lung pathology follows a varied pattern:

- diffuse alveolar damage (DAD) in different stages, up to the fibrotic stage which is usually associated with long-standing severe disease.
- infection of endothelial cells, edema, swelling, desquamation with phenomena of vascular thrombosis with remodeling of the vascular wall;
- possibility of secondary, bacterial and fungal infections;
- ventilator-induced lung injury (VILI) in patients undergoing prolonged period of assisted ventilation.

In the scientific panorama, there are not many studies that report pulmonary pathological patterns of the new Omicron virus variant. A recent published article [7] summarized data from 23 complete and 3 partial autopsies in deceased with known Omicron BA.1 and BA.2 infections. Despite high viral loads in nearly all nasopharyngeal swabs, death from COVID-19-associated diffuse alveolar damage (DAD) in the acute and organizational phases was found in only eight cases (31%). This rate was significantly lower than in previous studies, including non-Omicron variants. The study concluded that the mechanism leading to severe clinical outcome with major lung injury remains unclear and further studies including analysis of the host genome may be needed.

An actual open question is the pathological role of viral variants and the mechanism of organ damage [8]. All viruses, including SARS-CoV-2, change over time; and some changes may affect the properties of the virus, such as the ease with which it spreads, the severity of the associated disease or the performance of vaccines, therapeutic drugs, diagnostic tools or other health and social measures [9]. Although the new variants can (incorrectly) be categorized as "upper respiratory viruses", it has been seen that Omicron infection can easily lead to hospitalization, which is an important risk factor for the course of the disease. In fact, a long hospital stay increases the risk of coinfections or superinfections, with serious involvement of the lower respiratory tract. The autopsy activity, to date, represents an excellent tool for understanding the cytopathological processes that characterize the disease, providing a basis for clinical management.

The extent and severity of pulmonary complications solely related to the Omicron variant have not been fully determined. A key issue in all SARS-CoV-2 variants is whether the organ damage is due to direct organ targeting of the virus or downstream effects such as an alterated immune response. SARS-CoV-2 detection in tissues by Quantitative reverse transcription PCR (RT-qPCR) and immunohistochemistry (IHC) or electron microscopy (EM) can provide more details [10] on the matter.

Recent studies have shown that to reduce syncytial development in infected cells, Omicron changes the mechanism of fusion activation to the host cell; it lowers the chances of recognition through cell-to-cell communication [11]. The immune escape process occurs during the evolution process of the virus and helps the virus in its survival: viruses persist with lower lethality and a high spread rate, posing a significant risk to fragile patients and notably those immunosuppressed. What we are learning is that people with underlying conditions, people with advanced age, people who are unvaccinated can have a severe form of COVID-19 following Omicron variant infection [12].

The endpoint of future studies should be the impact of viral entry on immune evasion making Omicron the most significant variant of concern to date. The effect of Omicron spike mutations on domain-specific functions for antibody evasion, impaired cell–cell fusion, endosomal entry, and proteolytic processing needs to be investigated, providing a key resource for new therapeutic approaches [13].

Although the SARS-CoV-2 Omicron variant would appear to be associated with minor and less severe changes in chest CT images than previous variants [14], the immune escape process could increase hospitalization times in compromised patients and expose them at risk of pulmonary co-infections/superinfections.

From the limited literature data it is evident that a predominance of the direct effects of the viral infection would determine the disease immediately, while the indirect effects of the endothelial activation linked to the development of Acute Respiratory Distress Syndrome (ARDS) take over after a certain time of evolution of the disease. Given the greater tropism for the upper airways, it would be essential to minimize the factors involved in the perpetuation of the cycle that leads to persistence of lung damage, prolonged inflammatory response and fibroproliferation.

Dysregulation in neutrophil extracellular trap (NET) formation and degradation may play a role in the pathogenesis and severity of COVID-19. NETs have been detected in many organs of adult patients who died from complications of COVID-19 disease. However, infection with the Omicron variant would be associated with reduced "NETosis" phenomena compared to other SARS-CoV-2 variants [15].

As already mentioned, findings of different studies showed that Omicron was more immune evasive than other SARS-CoV-2 variants. The results highlighted the impairment in humoral and S-specific T lymphocyte immune responses post-infection among mild COVID-19 convalescent patients due to antigenic variability of the variants [16]. Evaluation of the functional responses of CD4 + and CD8 + memory T cells demonstrated that reduced recognition of the Omicron peak regards mainly the CD8 + T cell compartment, potentially due to escape from HLA binding [17].

In this context, the role of comorbidities in the pathogenesis and evolution of Omicron infection must be particularly taken into account; it will certainly depend on the type of disease with lung involvement. It has been shown that Omicron-infected patients with comorbidities could prolong the viral shedding time (VST) [18]. Factors such as albumin, CD4/CD8 ratio, neutrophil-to-lymphocyte ratio (NLR), and eosinophil count are risk factors for prolonged VST in patients infected with Omicron variant. These factors could be effective markers he diagnosis, evaluation and monitoring of the prognosis of the disease [19].

Lastly, genomic variations can unpredictably influence the manifestation of the infection, including the severity of acute disease; therefore, it will be crucial to sequence the viral genome that differentiates each Omicron subvariant. Studies that detect Omicron variant in the lower airways should be performed in the next future, as well as comparing them with the results obtained from the other variants, to better investigate the mechanism of lung injury.

## Data Availability

Not applicable.

## Abbreviations

SARS-CoV-2: Severe Acute Respiratory Syndrome Coronavirus 2
VOC: Variants of concern
COVID-19: Corona Virus Disease 2019
TMPRSS2: Transmembrane Serine Protease 2
DAD: Diffuse alveolar damage
VILI: Ventilator-induced lung injury
RT-qPCR: Quantitative reverse transcription—Polymerase chain reaction
IHC: Immunohistochemistry
EM: Electron microscopy
CT: Computed tomography
ARDS: Acute respiratory distress syndrome
NET: Neutrophil extracellular trap
HLA: Human leukocyte antigen
VST: Viral shedding time
NLR: Neutrophil-to-lymphocyte ratio

## References

[1] Aleem A, Akbar Samad AB, Slenker AK (2022). Emerging Variants of SARS-CoV-2 And Novel Therapeutics Against Coronavirus (COVID-19). StatPearls.

[2] Essalmani R, Jain J, Susan-Resiga D (2022). Distinctive Roles of Furin and TMPRSS2 in SARS-CoV-2 Infectivity. J Virol.

[3] Torjesen I (2021). COVID-19: Omicron may be more transmissible than other variants and partly resistant to existing vaccines, scientists fear. BMJ.

[4] Kozlov M (2022). Omicron’s feeble attack on the lungs could make it less dangerous. Nature.

[5] Fraser BJ, Beldar S, Seitova A (2022). Structure and activity of human TMPRSS2 protease implicated in SARS-CoV-2 activation. Nat Chem Biol.

[6] Scendoni R, Gattari D, Cingolani M (2022). COVID-19 Pulmonary Pathology, Ventilator-Induced Lung Injury (VILI), or Sepsis-Induced Acute Respiratory Distress Syndrome (ARDS)? Healthcare Considerations Arising From an Autopsy Case and Miny-Review. Clin Pathol..

[7] Märkl B, Dintner S, Schaller T (2022). Fatal cases after Omicron BA.1 and BA.2 infection: Results of an autopsy study. Int J Infect Dis..

[8] Jonigk D, Werlein C, Acker T (2022). Organ manifestations of COVID-19: what have we learned so far (not only) from autopsies?. Virchows Arch.

[9] Furuse Y (2022). Properties of the Omicron Variant of SARS-CoV-2 Affect Public Health Measure Effectiveness in the COVID-19 Epidemic. Int J Environ Res Public Health.

[10] Krasemann S, Dittmayer C, von Stillfried S (2022). Assessing and improving the validity of COVID-19 autopsy studies - A multicentre approach to establish essential standards for immunohistochemical and ultrastructural analyses. EBioMedicine.

[11] Willett BJ, Grove J, MacLean OA (2022). SARS-CoV-2 Omicron is an immune escape variant with an altered cell entry pathway. Nat Microbiol.

[12] Chakraborty C, Sharma AR, Bhattacharya M, Lee SS (2022). A Detailed Overview of Immune Escape, Antibody Escape, Partial Vaccine Escape of SARS-CoV-2 and Their Emerging Variants With Escape Mutations. Front Immunol.

[13] Omicron: a shift in the biology of SARS-CoV-2. Nat Microbiol. 2022;7(8):1114–1115. 10.1038/s41564-022-01149-1. 10.1038/s41564-022-01149-1 35821416

[14] Tsakok MT, Watson RA, Saujani SJ (2023). Reduction in Chest CT Severity and Improved Hospital Outcomes in SARS-CoV-2 Omicron Compared with Delta Variant Infection. Radiology.

[15] Carmona-Rivera C, Zhang Y, Dobbs K (2022). Multicenter analysis of neutrophil extracellular trap dysregulation in adult and pediatric COVID-19. JCI Insight.

[16] Garcia-Valtanen P, Hope CM, Masavuli MG (2022). SARS-CoV-2 Omicron variant escapes neutralizing antibodies and T cell responses more efficiently than other variants in mild COVID-19 convalescents. Cell Rep Med.

[17] Naranbhai V, Nathan A, Kaseke C (2022). T cell reactivity to the SARS-CoV-2 Omicron variant is preserved in most but not all individuals. Cell.

[18] Pei L, Chen Y, Zheng X (2022). Comorbidities prolonged viral shedding of patients infected with SARS-CoV-2 omicron variant in Shanghai: A multi-center, retrospective, observational study. J Infect Public Health.

[19] He S, Fang Y, Yang J (2022). Association between immunity and viral shedding duration in non-severe SARS-CoV-2 Omicron variant-infected patients. Front Public Health.

